# Non-Destructive Geographical Traceability and Quality Control of *Glycyrrhiza uralensis* Using Near-Infrared Spectroscopy Combined with Support Vector Machine Model

**DOI:** 10.3390/foods15030411

**Published:** 2026-01-23

**Authors:** Anqi Liu, Zibo Meng, Jiayi Ma, Jinfeng Liu, Haonan Wang, Yingbo Li, Yu Yang, Na Liu, Ming Hui, Dandan Zhai, Peng Li

**Affiliations:** 1 Henan Provincial Key Laboratory of Grain Resources Conservation and Utilization, School of Biological Engineering, Henan University of Technology, Zhengzhou 450001, China; angel_liu1023@163.com (A.L.); 15503835165@163.com (Z.M.); 18310822263@163.com (J.M.); ybli@ipe.ac.cn (Y.L.); liuna3456@163.com (N.L.); huiming@haut.edu.cn (M.H.); 2 Institute for Complexity Science, Henan University of Technology, Zhengzhou 450001, China; 17839093005@163.com (J.L.); whn1234560708@163.com (H.W.); yangxiangyu1168@haut.edu.cn (Y.Y.); 3 School of International Education, Henan University of Technology, Zhengzhou 450001, China; 4 College of Information Science and Engineering, Henan University of Technology, Zhengzhou 450001, China; 5 CAS Key Laboratory of Green Process and Engineering, Institute of Process Engineering, Chinese Academy of Sciences, Beijing 100190, China

**Keywords:** *Glycyrrhiza uralensis* Fisch., origin traceability, near-infrared spectroscopy, machine learning, spectral preprocessing

## Abstract

Licorice (*Glycyrrhiza uralensis* Fisch.) is a widely used natural sweetener and functional food ingredient. Its sensory profile, nutritional value, and bioactive composition are strongly affected by geographical origin and cultivation mode, particularly the distinction between wild and cultivated resources. Consequently, developing a rapid and robust method for origin traceability is imperative for rigorous quality control and product standardization. This study proposes a non-destructive traceability framework integrating near-infrared (NIR) spectroscopy with a Support Vector Machine (SVM). The method’s validity was rigorously evaluated using a comprehensive dataset collected from China’s three primary production regions—Gansu Province, the Inner Mongolia Autonomous Region, and the Xinjiang Uygur Autonomous Region, encompassing both wild and cultivated resources. Experimental results demonstrated that the proposed framework achieved an overall classification accuracy exceeding 99%. The results show that the proposed method offers a rapid, efficient, and environmentally friendly analytical tool for the quality assessment of licorice, providing a scientific basis for rigorous quality control and standardization in the functional food industry.

## 1. Introduction

Licorice (*Glycyrrhiza uralensis* Fisch.), is extensively utilized as a natural sweetener and functional food additive, and is officially recognized as a representative “medicine–food homology” resource [1]. Due to its unique sweetness and health-promoting properties, it is extensively utilized in beverages, confectionery, and dietary supplements [2,3,4]. These functional attributes are primarily attributed to its diverse bioactive constituents, including glycyrrhizic acid (a natural high-intensity sweetener) [5,6], liquiritin [7], flavonoids [8], and polysaccharides [9].

However, the accumulation of these nutritional and flavor-related metabolites is strictly governed by geographical and ecological conditions [10]; variations in climate, soil mineral content, altitude, and water availability across major producing regions such as Gansu Province (Gansu), the Inner Mongolia Autonomous Region (Inner Mongolia), and the Xinjiang Uygur Autonomous Region (Xinjiang) result in distinct metabolic patterns. Cultivation mode further contributes to compositional diversity [11], as wild licorice exposed to environmental stress (e.g., drought, salinity, and temperature fluctuation) tends to accumulate higher levels of secondary metabolites, whereas cultivated plants grown under managed agronomic conditions often exhibit more stable but sometimes lower constituent levels [12]. These origin and cultivation-dependent differences directly affect pharmacological efficacy and sensory quality, highlighting the need for rapid and reliable approaches to authenticate geographical origin and cultivation type even within the same botanical species.

Conventional analytical methods, including high-performance liquid chromatography (HPLC) [13], liquid chromatography–mass spectrometry (LC–MS), and ultraviolet spectrophotometry, provide accurate compositional information but are destructive, time-consuming, reagent-dependent, and unsuitable for large-scale or on-site monitoring. By contrast, near-infrared (NIR) spectroscopy offers a rapid, non-destructive, and environmentally friendly alternative capable of capturing overtones and combination bands of O–H, C–H, and N–H vibrations associated with major chemical constituents, thereby providing holistic chemical fingerprints without additional sample preparation [14,15]. Previous studies on American ginseng have demonstrated that NIR spectroscopy combined with chemometric or machine-learning methods can achieve accurate geographical discrimination, providing a methodological reference for licorice traceability research [16]. However, the high dimensionality, collinearity, and subtle variability inherent in NIR spectral data necessitate advanced computational tools for effective feature extraction and pattern recognition, making machine learning particularly suitable for this task [17].

To address the spectral complexity of biological matrices, researchers have increasingly employed deep learning architectures to mine latent non-linear patterns through hierarchical feature extraction [18]. However, deep learning typically requires massive datasets. In this study, the high-dimensional spectral features already exhibit sufficient separability to be resolved by classical algorithms. Therefore, we prioritized classical machine learning to adhere to the principle of model parsimony, achieving high accuracy without the complexity and overfitting risks of deep neural networks [19,20]. Among these classical approaches, the Support Vector Machine (SVM) stands out as a paradigmatic algorithm particularly well-suited for spectral analysis [21]. Its efficacy stems from the principle of structural risk minimization, which enables the construction of optimal separating hyperplanes to effectively resolve the high dimensionality and severe collinearity inherent in spectral data, ensuring robust performance even with moderate sample numbers [22]. Therefore, this study establishes a rapid and reliable authentication framework by combining NIR spectroscopy with machine learning to achieve the accurate and stable traceability of both geographical origin and cultivation mode of licorice.

The main contributions of this study are summarized as follows:

(1) A comprehensive NIR dataset was developed by non-destructively collecting spectral data of *Glycyrrhiza uralensis* samples from three major production regions (Gansu, Inner Mongolia, and Xinjiang) using a handheld NIR spectrometer (SW2960, OTO Photonics Inc., Hsinchu, Taiwan, China), covering both wild and cultivated resources.
(2) A systematic modeling framework was constructed by exhaustively optimizing spectral preprocessing techniques and comparing four machine learning algorithms (SVM, RF, kNN, and DT), demonstrating that the SVM model yields superior accuracy (>99%) and robustness for licorice traceability.

## 2. Materials and Methods

### 2.1. Sample Preparation and NIR Spectral Acquisition

In this study, a total of 1046 licorice samples, taxonomically authenticated as *Glycyrrhiza uralensis* Fisch., were collected from three major production regions in China: Gansu (GS), Inner Mongolia (NM), and Xinjiang (XJ). These locations represent the principal ecological zones for the growth of *G. uralensis*, where distinct differences in climate, soil, and environmental conditions significantly influence the accumulation of key bioactive constituents. To comprehensively reflect both environmental and cultivation variability, the dataset included a balanced distribution of resources: 167 cultivated and 104 wild samples from Gansu, 143 cultivated and 400 wild samples from Inner Mongolia, and 133 cultivated and 99 wild samples from Xinjiang. All samples were harvested in the autumn of 2024. The cultivated samples had a growth period of approximately three years. In contrast, the wild samples were influenced by environmental conditions, making their exact growth duration indeterminable. Consequently, they exhibited high heterogeneity, with some showing lignification or hollow structures. To facilitate an effective comparison with the cultivated samples, root diameters for both groups were selected within the range of 1.0 to 3.0 cm, which complies with the standard specifications of the Chinese Pharmacopoeia [23].

**Figure 1:**
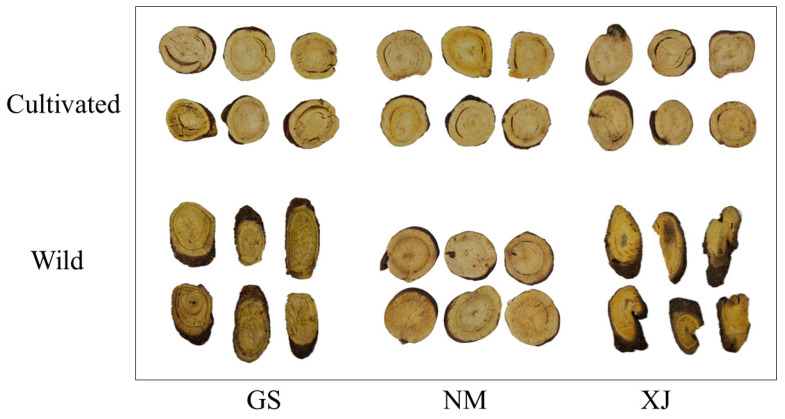
Representative photographs of licorice slices from different geographical origins and growth modes.

Prior to analysis, all samples were authenticated by a panel of experts from the Department of Pharmacy, Kaifeng Traditional Chinese Medicine Hospital. Fresh roots were processed into uniform sections (2–3 mm thickness) to ensure optical consistency, as illustrated in Figure 1, then dried at 50 °C and stored at 4 °C. To prevent the degradation of thermolabile components, samples were strictly maintained at low temperatures and only removed for equilibration immediately prior to spectral acquisition.

Spectral measurements were performed use a handheld NIR spectrometer (SW2960, OTO Photonics Inc., Hsinchu, Taiwan, China) covering the 900–2500 nm wavelength range with a resolution of 2 nm. Prior to usage, the instrument was preheated for 30 min and calibrated against a standard white ceramic reference to ensure baseline stability. For data acquisition, licorice slices were equilibrated to room temperature (≈25 °C) and positioned flat on the sample holder within a closed chamber to exclude ambient light. To account for potential surface heterogeneity, spectra were acquired in diffuse reflectance mode with three replicates per sample, involving slight probe repositioning between scans. The mean spectrum of these three replicates was utilized as the representative profile for each sample.

### 2.2. Data Processing and Model Establishment

#### 2.2.1. Dataset Partitioning and Spectral Preprocessing

The complete dataset, including cultivated and wild licorice samples collected from Gansu, Inner Mongolia, and Xinjiang, was divided into a training set and an independent test set at a ratio of 7:3. Sample partitioning was performed using the Kennard–Stone (KS) algorithm to ensure that the training set adequately covered the spectral variability of the full dataset. To further maintain class balance, stratified sampling was applied so that all six origin–cultivation categories were proportionally represented in both subsets.

All procedures related to model development, including spectral preprocessing optimization, hyperparameter tuning, and internal validation, were conducted exclusively on the training set. The independent test set was not involved in any stage of model construction and was reserved solely for the final assessment of model generalization performance.

**Figure 2:**
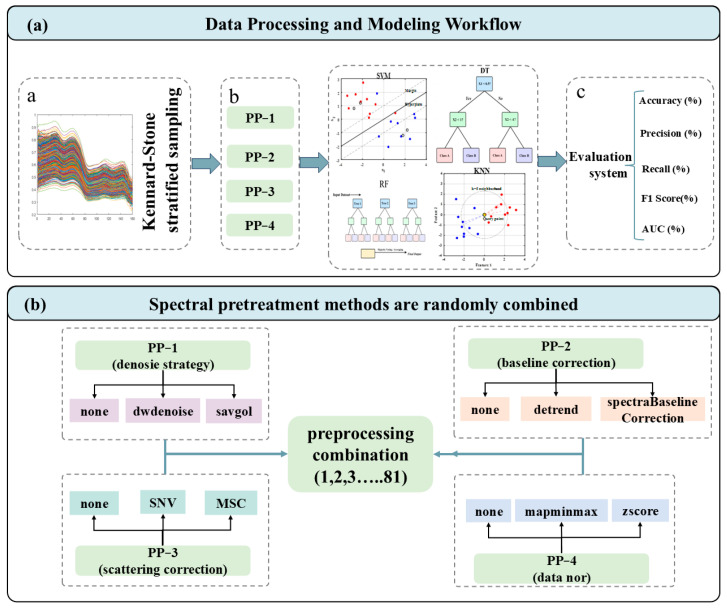
Framework of the proposed multi-task learning method. (**a**) The flowchart of the technical roadmap; (**b**) schematic of the stochastic preprocessing combination.

Raw NIR spectra are susceptible to noise, baseline drift, and light scattering effects associated with particle size variation and surface heterogeneity [24]. To address these issues in a systematic manner, an automated preprocessing evaluation scheme was established. As shown in Figure 2b, a total of 81 preprocessing combinations (3 × 3 × 3 × 3) were generated by permuting four preprocessing steps: denoising (None, Wavelet denoising, Savitzky–Golay smoothing), baseline correction (None, Detrending, Spectra Baseline Correction) [25], scatter correction (None, Standard Normal Variate (SNV), Multiplicative Scatter Correction (MSC)) [26], and normalization (None, Min–Max normalization (Mapminmax), Z-score normalization) [27].

#### 2.2.2. Model Construction

To achieve accurate geographical origin traceability and cultivation mode authentication, an integrated analytical workflow was implemented, encompassing spectral preprocessing, feature optimization, and classification modeling, as schematically illustrated in Figure 2a. This framework systematically evaluated the interaction between preprocessing pipelines and four classical machine learning classifiers: SVM, DT, kNN, and RF. The selection of these algorithms reflects a balance between computational simplicity, interpretability, and generalization ability for high-dimensional spectral data. Within the training set, a 5-fold cross-validation strategy was employed to optimize model hyperparameters and assess robustness. The training subset was randomly partitioned into five equal folds, with four folds used for training and one for validation in each iteration. This procedure was repeated so that each sample served once as the validation fold. The detailed hyperparameter settings for all models are listed in Table S2.

SVM was employed as the primary classifier due to its superior capability in handling high-dimensional and collinear spectral data based on the principle of structural risk minimization [28,29]. To address the non-linear separability of the complex spectral features, a Gaussian Radial Basis Function (RBF) kernel was utilized to map the input vectors into a higher-dimensional feature space. The RBF kernel function is defined as:

(1) $$K\left(x_{i},x_{j}\right)=\exp\left(-\gamma |x_{i}-x_{j}{|}^{2}\right)$$

where x*_i_* and x*_j_* are spectral vectors, and γ is the kernel scale parameter that controls the influence of a single training example. Consequently, the final classification decision function is determined by the weighted sum of support vectors:

(2) $$f\left(x\right)=\mathrm{sign}\left(\sum_{i=1}^{N}\alpha_{i}y_{i}K\left(x_{i},x\right)+b\right)$$

where N is the number of support vectors, α*_i_* are the Lagrange multipliers, y*_i_* denotes the class label, and b is the bias term. Both the penalty parameter C and the kernel parameter γ were optimized via grid search.

DT model was utilized to provide an interpretable classification structure, mapping spectral features directly to decision rules [30]. To mitigate the risk of overfitting inherent to high-dimensional spectral data, we implemented a pre-pruning strategy where the maximum depth of the tree was automatically determined to limit model complexity.

The tree construction employs a recursive partitioning approach. At each node, the feature (wavelength) that maximizes the purity of the split is selected. In this study, the Gini Impurity was used as the splitting criterion:

(3) $$Gini\left(D\right)=1-\sum_{k=1}^{K}p_{k}^{2}$$

where $p_{k}$ is the probability of a sample belonging to origin class k within the dataset D at the current node. The algorithm recursively minimizes the weighted sum of Gini impurities for the child nodes until the pruning criteria are met.

kNN is a non-parametric, instance-based learning algorithm that classifies samples based on local similarity in the spectral feature space [31]. It assumes that licorice samples with similar geographical origins share proximal positions in the high-dimensional space. In this study, the number of neighbors was fixed at k = 5, and the Euclidean distance was employed as the similarity metric [32].

For a query sample x and a training sample x_i, the distance is calculated as follows:

(4) $$d\left(x,x_{i}\right)=\sqrt{\sum_{j=1}^{n}{\left(x_{j}-x_{ij}\right)}^{2}}$$

The classification decision is made by majority voting among the k nearest neighbors:

(5) $$\hat{y}={\mathrm{argmax}}_{c}\sum_{i\in N_{k}\left(x\right)}I\left(y_{i}=c\right)$$

where $N_{k}\left(x\right)$ is the set of *k* nearest neighbors, c represents the class label, and $I\left(\cdot \right)$ is the indicator function.

RF is an ensemble learning algorithm that constructs a multitude of decision trees during training and outputs the class that is the mode of the classes of the individual trees [33]. It was employed to overcome the instability and high variance associated with single decision trees, making it particularly robust against noise and overfitting in high-dimensional NIR spectral data. In this study, the model was implemented with an ensemble of 50 trees using bootstrap aggregation (bagging) [34].

Mathematically, RF introduces randomness by resampling the training data with replacement and selecting a random subset of features at each split. The final predicted origin $\hat{y}$ for a licorice sample x is determined by majority voting across all T trees in the forest:

(6) $$\hat{y}={\mathrm{argmax}}_{c}\sum_{t=1}^{T}I\left(h_{t}\left(x\right)=c\right)$$

where h_t(x) represents the prediction of the t-th individual tree, and T=50. This ensemble approach ensures that the model remains robust even if individual trees are sensitive to specific spectral artifacts.

### 2.3. Performance Evaluation

The performance of the classification models was assessed using a comprehensive set of complementary metrics to ensure rigorous evaluation. While classification accuracy is widely reported, reliance on this single metric can be misleading, particularly under conditions of class imbalance. To overcome this limitation, Precision and Recall were calculated on a macro-averaged basis to evaluate the minimization of false positives and classifier sensitivity, respectively. To balance these dimensions, both macro- and weighted-average F1-scores were computed; the former treats all classes equally to assess balanced performance, while the latter accounts for differences in sample distribution. Furthermore, the Area Under the Receiver Operating Characteristic Curve (AUC) [35] was included as a threshold-independent measure of discriminative capability, which is particularly valuable for multi-class problems [36].

These metrics were computed within an integrated assessment workflow designed to standardize performance comparison across heterogeneous classifiers. By transforming classifier-specific outputs—such as probability estimates, distance-based measures, or ensemble voting results—into a unified representation, the workflow ensured dimensional consistency and verified the correctness of matrix operations throughout the iterative modeling process. All spectral preprocessing, machine learning implementation, and statistical analyses were performed using MATLAB R2019a.

## 3. Results

### 3.1. Spectral Analysis

**Figure 3:**
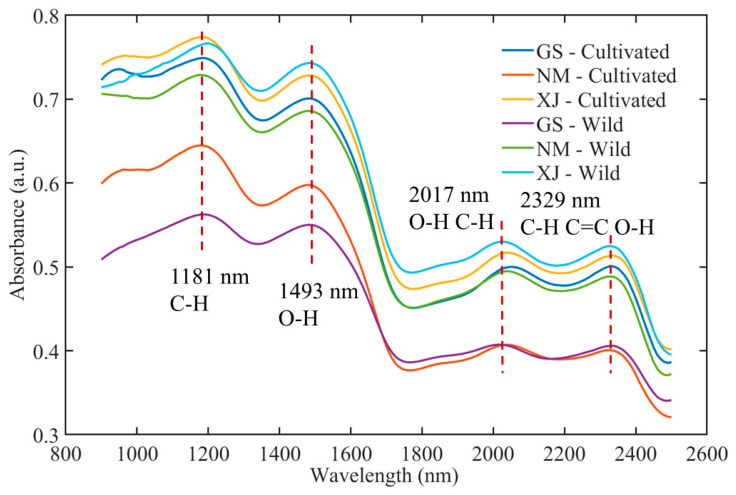
Average NIR spectra of licorice samples.

The raw NIR spectra of licorice samples collected from different geographical origins and cultivation types are presented in Figure 3. The spectra exhibited characteristic absorption bands associated with major functional groups, reflecting the complex chemical matrix of licorice [37]. Prominent absorption peaks were observed around 1181 nm (C–H stretching overtones), 1493 nm (O–H first overtone), 2017 nm (combination bands of O–H and C–H), and 2329 nm (C–H, C=O, and O–H combinations) [38]. These bands are closely correlated with key bioactive constituents, including saponins, flavonoids, and polysaccharides. While the general spectral profiles followed a consistent trend across all samples, variations in absorbance intensity and band sharpness were noted among origins (Gansu, Inner Mongolia, and Xinjiang) and growth modes (wild vs. cultivated), attributable to the influence of distinct ecological environments on metabolite accumulation [39].

**Figure 4:**
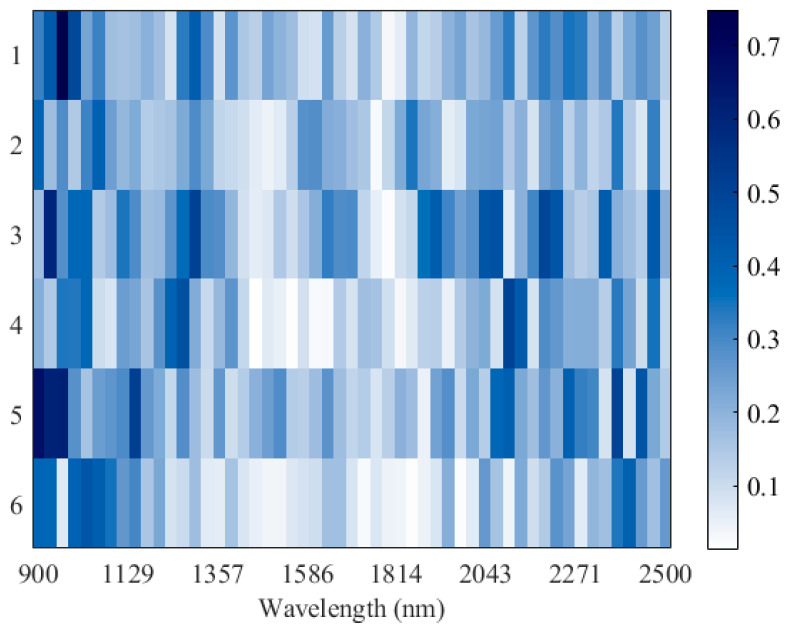
Heatmap of NIR spectra (900–2500 nm) for licorice samples. Labels 1–3 and 4–6 represent cultivated and wild samples from Gansu, Inner Mongolia, and Xinjiang, respectively. The color intensity indicates the magnitude of absorbance, with darker colors corresponding to stronger absorption.

To further visualize the spectral heterogeneity across the full wavelength range (900–2500 nm), a heatmap representation was generated (Figure 4). Labels 1–3 correspond to cultivated samples, while labels 4–6 denote wild samples from the three regions. The heatmap reveals that wild and cultivated licorice exhibit distinct patterns of light absorption, particularly in the spectral regions around 1350–1600 nm and 2000–2400 nm [40], which are consistent with water- and carbon-related vibrations. This intuitive visualization confirms that geographical and cultivation differences translate into discernible spectral variations [41].

**Figure 5:**
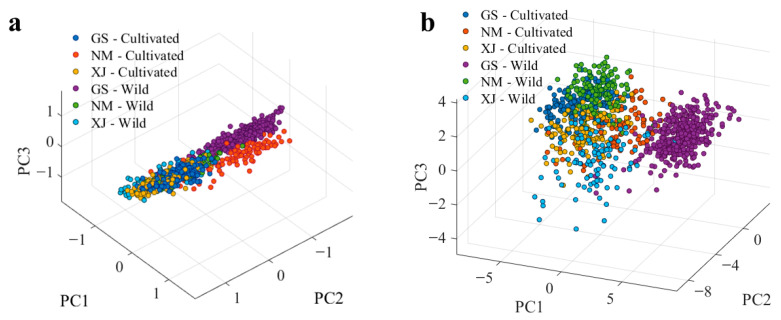
PCA score plots of the licorice samples. (**a**) Raw spectra; (**b**) preprocessed spectra.

However, direct visual inspection and raw spectral data are often insufficient for accurate discrimination due to the presence of high-frequency noise, baseline drift, and light scattering [42]. To quantitatively assess the intrinsic data structure and evaluate the efficacy of the proposed preprocessing workflow, Principal Component Analysis (PCA) was conducted [43]. As illustrated in the score plot of raw spectra (Figure 5a), substantial overlap was observed among samples from different origins and cultivation types. This lack of separation indicates that physical artifacts masked the subtle chemical variations, thereby limiting the direct applicability of machine learning to raw data.

In contrast, after applying the systematic preprocessing workflow, the PCA score plot (Figure 5b) exhibited markedly improved discrimination. Samples from Gansu, Inner Mongolia, and Xinjiang formed distinct, well-defined clusters, with clear sub-groupings observed between cultivated and wild resources. The transition from the disordered overlap in Figure 5a to the structured clustering in Figure 5b confirms that the preprocessing pipeline effectively suppressed spectral artifacts, improved the signal-to-noise ratio, and amplified chemically relevant features. These results establish a robust, high-quality data foundation for the subsequent development of supervised classification models.

### 3.2. Model Performance with Different Preprocessing

Table 1 summarizes the classification performance of the four machine learning models evaluated using a 5-fold cross-validation strategy. This validation approach was employed to rigorously assess model generalization and ensure that the high classification accuracy was not a result of overfitting. Among the evaluated classifiers, the SVM model demonstrated the most robust performance. It achieved a near-perfect mean OA of 99.81% ± 0.43%, with Precision, Recall, F1, and AUC values all exceeding 99.7%. The minimal standard deviation across the five folds indicates that the SVM, utilizing the RBF kernel, effectively resolved the overlapping absorption bands inherent in the complex herbal matrix while maintaining stability across independent data subsets.

The kNN and RF models also yielded satisfactory results, with mean accuracies exceeding 98% and 99%, respectively. The kNN model effectively leveraged local neighborhood structures, while the RF model benefited from ensemble averaging to reduce variance. However, statistical analysis (paired *t*-test) indicated that their stability was slightly lower than that of SVM. In contrast, the DT model exhibited the poorest performance (Mean OA = 87.10 ± 2.05%), reflecting the susceptibility of single-tree structures to noise and high dimensionality in NIR data.

The cross-validation results provide statistical evidence that the superior performance of the SVM model is attributed to its structural suitability for high-dimensional spectral data rather than overfitting.

### 3.3. Comparison of Classification Models

**Figure 6:**
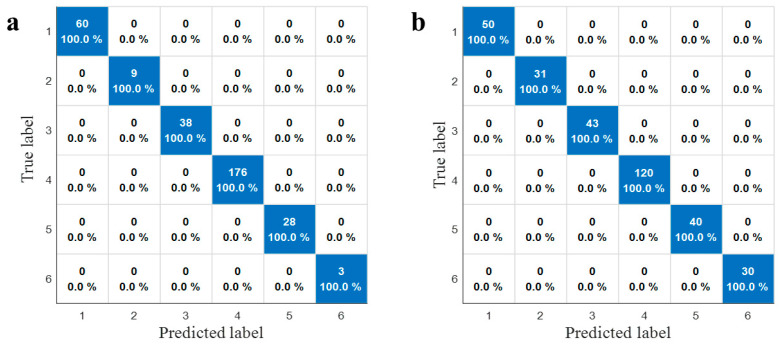
Confusion matrices of SVM models based on different data partitioning algorithms. (**a**) Kennard–Stone (KS) algorithm; (**b**) stratified sampling.

To validate the robustness of the classification models against dataset variations, the dataset was partitioned using two distinct strategies: the Kennard–Stone (KS) algorithm and stratified sampling (70:30 ratio). The reliability of the best-performing classifier was corroborated by the comparative confusion matrices of the SVM model shown in Figure 6. Remarkably, the SVM classifier exhibited high stability, yielding identical classification patterns under both partitioning strategies. As illustrated in Figure 6a (KS) and Figure 6b (Stratified sampling), the model achieved 100% accuracy across all six categories—including cultivated and wild licorice from Gansu, Inner Mongolia, and Xinjiang—without a single misclassification. This consistency confirms that the model’s performance is robust to different data splitting methods. Furthermore, model stability was substantiated through multiple randomized runs, where a low standard deviation in classification accuracy (<0.5%) demonstrated excellent reproducibility.

In the comprehensive evaluation of all classifiers, the SVM model emerged as the optimal classification strategy, demonstrating superior performance over other algorithms. While RF also yielded competitive results with accuracies exceeding 99%, SVM exhibited unmatched robustness in handling high-dimensional and collinear spectral data, achieving perfect classification in the validation phase. Its ability to maximize the decision margin allowed it to capture complex nonlinear relationships more effectively than kNN, which showed sensitivity to sample distribution, or DT, which suffered from overfitting. SVM was identified as the most reliable and effective tool for the precise traceability of licorice origin and cultivation type.

### 3.4. Chemical Composition Interpretation

**Figure 7:**
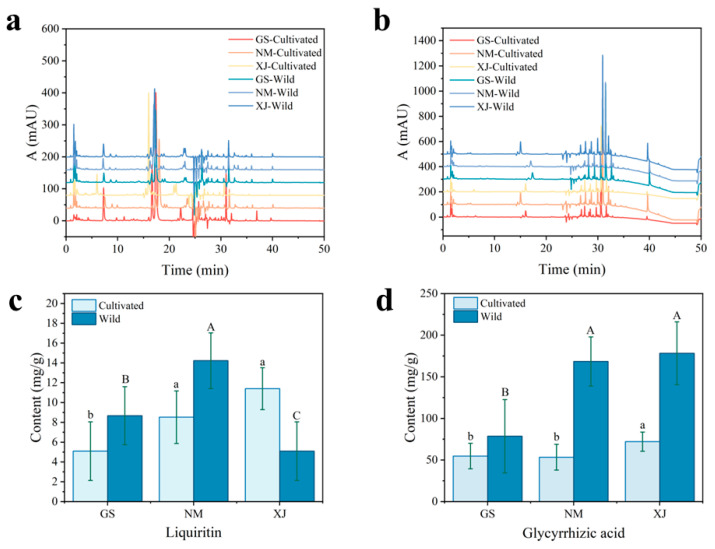
HPLC chromatograms and active ingredient contents of wild and cultivated licorice samples from different geographical origins. (**a**) Chromatograms at 274 nm (Liquiritin); (**b**) Chromatograms at 254 nm (Glycyrrhizic acid); (**c**) Content of liquiritin; (**d**) Content of glycyrrhizic acid. Data are expressed as mean ± SD (*n* = 75). Different lowercase letters (a, b) and uppercase letters (A, B) indicate statistically significant differences (*p* < 0.05) among cultivated and wild samples, respectively.

To elucidate the chemical basis underlying the spectral differentiation among licorice samples, HPLC was employed to quantify major bioactive constituents, specifically glycyrrhizic acid and liquiritin. These compounds were selected as reference markers due to their status as statutory quality indicators in the Chinese Pharmacopoeia [23], their dominance in content, and their role as core metabolites representing the triterpene saponin and flavonoid classes, respectively [44,45]. Representative chromatograms are shown in Figure 7a,b. Detailed quantitative results are provided in Table S1. As visualized in Figure 7c,d, the analysis revealed significant compositional heterogeneity driven by geographical factors, with cultivated licorice from Xinjiang exhibiting notably higher glycyrrhizic acid content compared to other origins.

These compositional variations provide a structural basis for the observed NIR spectral features. Glycyrrhizic acid is characterized by a triterpenoid skeleton rich in C–H bonds and two glucuronic acid moieties containing abundant O–H groups, while liquiritin features a flavonoid backbone with phenolic hydroxyls and a glucose unit [46]. These structural moieties directly correspond to the dominant NIR absorption bands. The strong absorbance in the 1400–1600 nm region (O–H first overtone) arises from the hydroxyl groups in the sugar moieties and phenolic rings. Similarly, signals in the 2100–2300 nm region are attributed to the combination bands of C–H stretching vibrations from the carbon skeletons and O–H/C–O vibrations from the glycosidic structures [47].

Correlation analysis confirmed strong linear dependencies between HPLC-determined concentrations and spectral intensities at characteristic wavelengths, such as 1180 nm (C–H second overtone of the aliphatic skeleton) and 1490 nm (O–H first overtone) [48]. Although only two markers were quantified, the high classification accuracy suggests that the machine learning models utilized the holistic spectral fingerprint. This comprises not only glycyrrhizic acid and liquiritin but also the co-varying matrix components, thereby capturing the comprehensive metabolomic phenotype rather than relying on isolated chemical markers.

## 4. Discussion

The present study establishes that the integration of NIR spectroscopy with machine learning offers a distinct methodological advantage for tracing the geographical origin and cultivation type of *Glycyrrhiza* spp. Unlike conventional chromatography (HPLC), which necessitates destructive and reagent-intensive preparation, the proposed spectral strategy enables expeditious analysis while retaining holistic chemical information [49]. Specifically, the spectral fingerprints capture O–H, C–H, and N–H vibrational modes corresponding to the comprehensive matrix of saponins, flavonoids, and polysaccharides, rather than quantifying isolated markers [50]. Furthermore, in contrast to genomic approaches that delineate genetic lineage, NIR spectroscopy characterizes the phenotypic expression shaped by the interaction between genotype and environmental factors (edaphic and climatic conditions), thereby providing a more direct representation of quality-related differentiation [51].

Regarding classification performance, the systematic evaluation of preprocessing–model interactions substantiates the superiority of SVM and RF over simpler classifiers (DT, kNN). The robustness of SVM is attributed to its structural risk minimization principle, which effectively manages high-dimensional collinear spectral data by optimizing decision boundaries. Conversely, the efficacy of RF derives from ensemble learning, which mitigates variance and reduces the risk of overfitting inherent in single decision trees [32]. These findings align with prior chemometric studies on *Panax ginseng* and *Angelica sinensis*, reinforcing the validity of data-driven approaches for authenticating medicinal-food homology materials [52].

Despite these promising laboratory results, several methodological constraints must be critically addressed to assess their real-world applicability. The current study relies on a static dataset acquired under controlled conditions. The absence of independent validation on external datasets spanning multiple harvest years or diverse storage environments restricts the assessment of the model’s temporal generalization [53]. Without such validation, the high classification accuracy observed may partially reflect sampling bias linked to the specific spatiotemporal context. In addition, instrument-to-instrument variability represents a significant source of uncertainty when deploying laboratory models in practical settings. Differences in detector type, light source, wavelength calibration, and optical geometry can introduce spectral shifts and baseline distortions, potentially reducing prediction accuracy and model transferability [54].

The high classification accuracy (100%) achieved by SVM in this study is partly attributed to the standardized preparation of licorice slices, which minimized the interference of sample morphology [55]. However, the current model has certain limitations. SVM and other shallow learning algorithms primarily focus on global spectral features, which limits their ability to decouple non-linear physical interferences—such as varying sample thickness, moisture content, and surface residues—from the intrinsic chemical absorption signals. To address this, future work must move beyond classical machine learning; 1D-CNNs are essential not as a speculative upgrade, but as a robust tool to model spectral distortions arising from physical heterogeneity. Additionally, to resolve the barrier to scalability caused by instrument-to-instrument variability, Transfer Learning is required to correct domain shifts arising from hardware-specific spectral shifts. This ensures that the high-accuracy model developed on a master device can be reliably deployed across diverse instrumental and environmental platforms [56].

## 5. Conclusions

This study validates the feasibility of employing NIR spectroscopy combined with machine learning algorithms for the non-destructive traceability of licorice across the geographical origins of Gansu, Xinjiang, and Inner Mongolia, covering both wild and cultivated resources. By systematically evaluating multiple spectral preprocessing techniques alongside four classical classifiers, we established a robust analytical framework where SVM and RF consistently outperformed DT and kNN, achieving classification accuracies exceeding 90% across all pipelines. Specifically, SVM exhibited the highest stability and precision, making it ideal for strict quality enforcement, while RF demonstrated strong robustness against the noise and sample variability typical of complex biological matrices. A notable advantage of the proposed strategy lies in the use of a comprehensive multi-metric evaluation scheme (incorporating Accuracy, Precision, Recall, F1-score, and AUC) rather than relying solely on accuracy, ensuring that the model possesses the generalization capability required for routine industrial monitoring. Collectively, these findings indicate that NIR spectroscopy, when coupled with robust classifiers like SVM, offers a rapid, reliable, and “green” approach for authenticating the quality and consistency of licorice resources, holding significant potential for broader application in the traceability and standardization of functional food ingredients to support the integrity and transparency of the global food supply chain.

## Figures and Tables

**Table 1:** Classification performance of different models using 5-fold cross-validation.

Model	Preprocessing	OA (%)	P (%)	R (%)	F1 (%)	AUC (%)
SVM	0-2-0-1	99.81 ± 0.43	99.92 ± 0.19	99.70 ± 0.67	99.81 ± 0.43	100 ± 0.00
DT	2-2-0-0	87.10 ± 2.05 **	84.20 ± 2.45 **	84.08 ± 3.06 **	83.77 ± 2.76 **	92.87 ± 2.15 **
kNN	0-2-0-2	98.09 ± 3.49 *	98.59 ± 3.01	97.06 ± 6.04 *	97.73 ± 4.54 *	99.83 ± 0.39
RF	0-2-0-1	99.23 ± 0.66 *	99.46 ± 0.54 *	98.78 ± 1.15*	99.09 ± 0.88 *	99.82 ± 0.20

Note: N-N-N-N denotes the number of the spectra denoising, baseline drift, scattering correction, and scaling used; Data represent mean ± SD from 5 independent folds. Statistical analysis was performed using a paired *t*-test comparing each model against SVM. * indicates *p* < 0.05 and ** indicates *p* < 0.01.

## Data Availability

The original contributions presented in this study are included in the article/Supplementary Materials. Further inquiries can be directed to the corresponding authors.

## Supplementary Materials

The following supporting information can be downloaded at: https://www.mdpi.com/article/10.3390/foods15030411/s1, Table S1: The contents of glycyrrhizin and glycyrrhizic acid in wild and cultivated licorice from different origins; Table S2: Hyperparameter settings for the machine learning models employed in this study.

## Abbreviations

The following abbreviations are used in this manuscript:

NIR	Near-Infrared
SVM	Support Vector Machine
Gansu	Gansu Province
Inner Mongolia	Inner Mongolia Autonomous Region
Xinjiang	Xinjiang Uygur Autonomous Region
HPLC	High-performance liquid chromatography
LC-MS	liquid chromatography–mass spectrometry
RF	Random Forest
DT	Decision Tree
kNN	k-Nearest Neighbors
KS	Kennard–Stone
SNV	Standard normal variate
MSC	Multiplicative scatter correction
AUC	Area Under the Receiver Operating Characteristic Curve
PCA	Principal Component Analysis
OA	Overall Accuracy
RBF	Radial Basis Function
