# Supplementary material for: Non-Destructive Geographical Traceability and Quality Control of Glycyrrhiza uralensis Using Near-Infrared Spectroscopy Combined with Support Vector Machine Model
Source: Foods. 2026 Jan 23;15(3):411. doi: 10.3390/foods15030411 (PMC12896434; doi:10.3390/foods15030411)
Supplement: Supplementary file 1 [file foods-15-00411-s001.zip › foods-4086661-supplementary.pdf]

Supporting Information

For

Non-destructive geographical traceability and quality control of Glycyrrhiza uralensis using near-infrared spectroscopy combined with machine learning models

Submitted to

Foods

S1. HPLC analysis method

S1.1. Sample preparation and extraction

Prior to chemical analysis and following NIR spectral acquisition, G. uralensis root samples were finely pulverized and sieved through an 80-mesh screen to ensure homogeneity. An aliquot of 100 mg of the powdered sample was accurately weighed for extraction. The extraction process was carried out using 3 mL of aqueous solution (pH 7.9) under ultrasonic conditions (40 kHz, 100 W) at 80 °C for 47 min. Subsequently, the extracts were centrifuged at 12,000 rpm for 15 min at 25 °C. The supernatant was filtered through a 0.45 μm microporous membrane prior to HPLC injection.

S1.2. Chromatographic conditions

Chromatographic separation was performed using an Agilent 1260 Infinity LC system (Agilent Technologies, USA). The separation was achieved on an Elite AQC18 column (4.6 × 150 mm, 5 μm; Dalian Elite Analytical Instruments Co., Ltd., China). The mobile phase consisted of acetonitrile (A) and water (B) using the following gradient elution program: 0 – 5 min, 8% A; 5 – 20 min, 8 – 18% A; and 20 – 32 min, 18 – 50% A. The flow rate was maintained at 1.0 mL/min, the column temperature was set to 30 °C, and the injection volume was 10 μL. The detection wavelengths were set at 280 nm for liquiritin and 250 nm for glycyrrhizic acid.

Table S1 The contents of glycyrrhizin and glycyrrhizic acid in wild and cultivated licorice from different origins

Place of origin	Cultivated or wild	Glycyrrhizin content (mg/g)	Glycyrrhizic acid content (mg/g)
Gansu Province	Cultivated	5.09 ± 2.96	54.75 ± 15.32
	Wild	8.67 ± 2.93	78.55 ± 44.04
Inner Mongolia	Cultivated	8.52 ± 2.65	53.31 ± 15.47
Autonomous Region	Wild	14.22 ± 2.8	168.36 ± 29.54
Xinjiang Uygur	Cultivated	11.4 ± 2.11	72.05 ± 11.54
Autonomous Region	Wild	5.09 ± 2.96	178.27 ± 37.73

Note: Data are expressed as mean ± SD (n=75).

Table S2 Hyperparameter settings for the machine learning models employed in this study

Classifier	Hyperparameter	Optimal Value
SVM	Kernel Function	Gaussian (RBF)
	Box Constraint (C)	10
	Kernel Scale (γ)	0.1
DT	Max Depth	50
	Min Samples per Leaf	5
	Min Samples per Split	5
kNN	Number of Neighbors (k)	3
	Distance Metric	Euclidean
	Distance Weighting	Distance
	Distance Power (p)	2
RF	Number of Trees	200
	Max Depth	50
	Min Samples per Leaf	5
	Max Features	sqrt
